# Bumblebees Use Sequential Scanning of Countable Items in Visual Patterns to Solve Numerosity Tasks

**DOI:** 10.1093/icb/icaa025

**Published:** 2020-05-05

**Authors:** HaDi MaBouDi, H Samadi Galpayage Dona, Elia Gatto, Olli J Loukola, Emma Buckley, Panayiotis D Onoufriou, Peter Skorupski, Lars Chittka

**Affiliations:** School of Biological and Chemical Sciences, Queen Mary University of London, London E1 4NS, UK; School of Biological and Chemical Sciences, Queen Mary University of London, London E1 4NS, UK; Department of General Psychology, University of Padova, 35131 Padova, Italy; School of Biological and Chemical Sciences, Queen Mary University of London, London E1 4NS, UK; School of Biological and Chemical Sciences, Queen Mary University of London, London E1 4NS, UK; Faculty of Biology, Medicine and Health, University of Manchester, Manchester M13 9PL, UK; School of Biological and Chemical Sciences, Queen Mary University of London, London E1 4NS, UK; Institute of Medical and Biomedical Education, St George’s, University of London, London SW17 0RE, UK; School of Biological and Chemical Sciences, Queen Mary University of London, London E1 4NS, UK; Wissenschaftskolleg zu Berlin—Institute for Advanced Study, Wallotstrasse 19, 14193 Berlin, Germany

## Abstract

Most research in comparative cognition focuses on measuring *if* animals manage certain tasks; fewer studies explore *how* animals might solve them. We investigated bumblebees’ scanning strategies in a numerosity task, distinguishing patterns with two items from four and one from three, and subsequently transferring numerical information to novel numbers, shapes, and colors. Video analyses of flight paths indicate that bees do not determine the number of items by using a rapid assessment of number (as mammals do in “subitizing”); instead, they rely on sequential enumeration even when items are presented simultaneously and in small quantities. This process, equivalent to the motor tagging (“pointing”) found for large number tasks in some primates, results in longer scanning times for patterns containing larger numbers of items. Bees used a highly accurate working memory, remembering which items have already been scanned, resulting in fewer than 1% of re-inspections of items before making a decision. Our results indicate that the small brain of bees, with less parallel processing capacity than mammals, might constrain them to use sequential pattern evaluation even for low quantities.

## Introduction

Numerical cognition is viewed as a hallmark of higher cognitive abilities and intelligence in animals, perhaps because of the perceived association between mathematical competence and intelligence in humans (Dehaene 2011). Numerical abilities have been found in primates (Brannon and Terrace 2000), birds (Rugani et al. 2013), amphibians (Uller et al. 2003), fish (Agrillo et al. 2012), and some invertebrates (Chittka and Geiger 1995; Dacke and Srinivasan 2008; Gross et al. 2009; Carazo et al. 2012; Yang and Chiao 2016; Howard et al. 2018), but few studies have explored the animals’ pattern inspection tactics by which such tasks are solved. This may be partly because the researchers’ goal was often to demonstrate animal intelligence, in which case it perhaps appears sufficient to measure performance and be satisfied that the animal is successful in a statistically supportable manner. However, seemingly complex cognitive tasks can sometimes be solved by elegantly simple shortcuts (Guiraud et al. 2018), using very basic neural circuitry (MaBouDi et al. 2017; Peng and Chittka 2017; Roper et al. 2017). We therefore think it is imperative to explore the behavioral strategies by which animals solve cognitive tasks, in addition to testing whether or not they solve them (Skorupski et al. 2018; Vasas and Chittka 2019).

Studies on adult and infant humans and a variety of other species have suggested the existence of two number systems: a small number system, which represents the numerosity of sets of up to four items, and a large number system, which represents the approximate numerosity of larger sets, but with an error that scales with set size (Trick and Pylyshyn 1993; Pylyshyn 2001; Burr et al. 2010; Hyde 2011; Skorupski et al. 2018). The ability of humans and at least some other primates to accurately perceive small numerosities “at a glance” has been termed subitizing (Jevons 1871; Kaufmann et al. 1949; Matsuzawa 2009). Comparative studies have led to the hypothesis that the small and accurate number system (object file system or OFS) and large but approximate (analog magnitude) number systems rest upon mechanisms shared by a variety of species (approximate number system or ANS) (Feigenson et al. 2004). The comparative evidence for this mainly comes from studies showing a discontinuity in performance, where error rates are relatively constant for set sizes of up to four, but increase with set size for larger numbers of items (Weber’s law). However, the existence of such a discontinuity has been challenged in non-human and human studies (Rugani et al. 2013). However, the OFS is thought to depend on object perception and individuation, which depends on working memory and which would also explain the upper limit for this system of three to four items (Cowan 2001). Even though there is also evidence to suggest that performances seen for small versus large numbers might be underpinned by a single system (Gallistel 1990; Dehaene and Brannon 2011; Halberda and Odic 2015; Cheyette and Piantadosi 2020), there is no controversy about the observation that humans and some other animals are exceptionally fast and accurate at assessing numbers of up to four. The ability to process visual information rapidly and in parallel appears to be a general feature of the primate visual system.

Bees, on the other hand, appear poorly able to analyze entire visual scenes at a glance (Nityananda et al. 2014; Guiraud et al. 2018) and this might also be reflected in their counting performance. Honeybees can discriminate visual patterns with small numbers of items based on numerical cues (Gross et al. 2009; Howard et al. 2018). We hypothesize that bees will be unable to rely on a single sensory snapshot to make numerical discriminations and predict instead that enumeration of small sets of items will be dependent on sequential scanning (Skorupski et al. 2018). This implies that the time required to make number-based visual discriminations will depend on the set sizes to be enumerated. Here, we explore this prediction by detailed analysis of the behavior of bees during the decision-making process in a numerosity discrimination task.

## Methods

### Bees and apparatus

Eight colonies of bumblebees (*Bombus terrestris audax*) were used in this study, housed in individual nest-boxes. Each nest was separately connected to a wooden flight arena (100 × 70 × 70 cm) via a plastic tunnel. The arena was covered with a UV-transparent Plexiglas ceiling.

Prior to experiments, a gravity feeder containing 30% sucrose solution was placed in the center of the arena to familiarize with the experimental arena. In this stage, forager bees could freely return to the hive when satiated. Successful foragers were individually marked on the thorax with number labels for identification during the subsequent experiment.

Marked bees were initially pre-trained to receive 50% sucrose solution from 10 white disks (7 cm in diameter) surrounded by 2 mm wide black margins presented on the back wall of the arena. The center of each disk was attached to the back wall of the arena via a tube (5 mm in diameter); a drop of 50% sucrose solution was placed in the opening of the tube in the center of the disk. Foragers that learned to take the sucrose from the center of the pattern were selected for the experiment.

### Stimuli

**Fig. 1:**
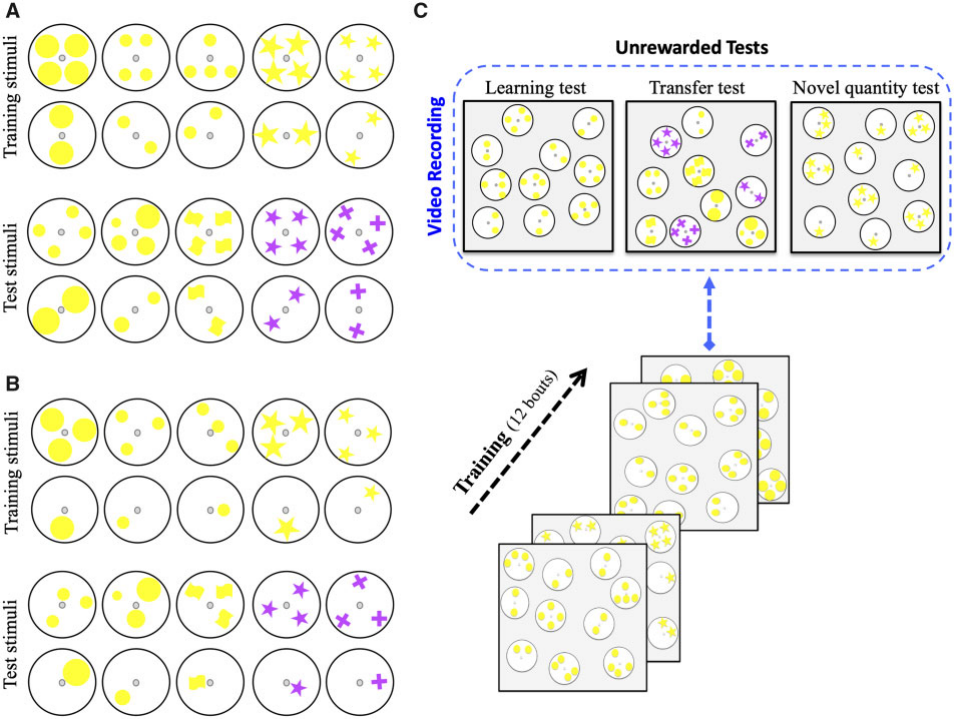
Training and testing protocol. (**A**, **B**) Training and test patterns (artificial flowers) were constructed from 7 cm diameter disks with a variable number (1–4) of constituent elements differing in size and shape (small or large circles or stars). Test patterns included the same stimuli used during training, and additionally, during transfer tests, stimuli whose constituent elements contained novel shapes, size, and color. Each pattern was attached via its center to the rear wall of the flight arena by a plastic tube (5 mm diameter) with 10 μL sucrose or quinine (training) or distilled water (test stimuli) placed at the opening. Patterns were rotated around the center during the experiment, to vary orientation of the pattern elements in a pseudo-random manner. Bees were trained on patterns containing either one or three elements (A), or two or four elements (B); in each case differential conditioning was used, in separate groups of bees, such that either the higher or lower numerosity was positively reinforced (sucrose) and the complementary numerosity was negatively reinforced (quinine). (**C**) Each bee was subjected to 12 training bouts, in which she entered the flight arena and was confronted with five pairs of patterns (e.g., two-item versus four-item patterns). The bee was free to sample the rewarding and unrewarding patterns and return to the nest box when satiated, which marked the end of a bout. Following training bees were subjected to three further tests where the positive or negative reinforcement was replaced with sterile distilled water. Responses were analyzed from video recording of the first 120 s in the flight arena. In learning tests, bees were presented with pattern pairs randomly selected from the training set. In transfer tests bees were presented with patterns of the same numerosity but with constituent elements of novel size, shape, and color. Finally, bees were confronted with novel numerosity tests, such that bees trained to discriminate one- from three-item patterns were presented with two- versus four-item patterns, and vice versa.

Stimulus patterns were constructed from the same 7 cm white disks, but with a varying number of constituent elements to vary numerosity. These consisted of two yellow shapes (circles or stars) in two different sizes (3.1 and 7.0 cm^2^). The number of items in a pattern was one, two, three, or four, each presented in one of four alternative configurations (small or large circles or stars). Patterns were rotatable about their centers to vary pattern orientation between training bouts and between tests. Each pattern was surrounded by a 2 mm wide black margin, and subtended a visual angle of approximately 4.5° from the entrance to the flight arena. Patterns were laminated to allow cleaning between training bouts and tests. Patterns were presented in alternative numerosity pairs: either one-item and three-item patterns, or two-item and four-item patterns (see Fig. 1A, B for some training patterns). Additional patterns were constructed for transfer tests (see below). These were designed in a different size such that the total yellow color area in the pattern with larger number of items was less than the total yellow color in the alternative pattern. Other patterns were constructed using a novel color (purple) and novel shapes (Fig. 1A, B). We measured the spectral reflectance of the yellow and purple items as well as the white background against which they were presented, following methods by Chittka (1992) and using the spectral sensitivity functions of the bumblebee *B. terrestris* (Skorupski et al. 2007). The lab’s illumination spectrum was taken from Li et al. (2017). We calculated the receptor signals in the bees’ UV, blue and green receptors for the countable items. From these values we calculated the color contrast and green contrast (contrast perceived by the bees’ green receptors), since both can be used alternatively in stimulus detection by bumblebees (Spaethe et al. 2001; Dyer et al. 2008). Yellow targets produced a high color contrast against their white background (0.32). A color contrast of >0.3 has been empirically shown to result in very high levels of detectability and minimal search times in bumblebees (Spaethe et al. 2001). Yellow and purple targets differed by a Euclidian distance of 0.31 in the bee color space, where values of 0.2 already result in close to 100% accuracy in color discrimination (Dyer and Chittka 2004). Green contrast for the yellow targets was low (0.01), but this is more than compensated for by high values of color contrast for these targets (note that unlike honeybees, bumblebees can use color contrast for target detection even in the absence of green contrast [Dyer et al. 2008]). Green contrast for the purple items with the white background was −0.13. See Supplementary Table S1 for further details on stimulus parameters as the edge length, total amount of color, special frequency, convex hull, and illusionary shape of stimuli.

### Protocol

During the training phase, the rear wall of the arena served as a decision wall. Five pairs of disks were randomly placed, presenting two alternative patterns, and differential conditioning was used to improve decision accuracy. Positive reinforcement was provided by 10 μL 50% sucrose solution placed at the center of the target pattern and negative reinforcement by 10 μL saturated quinine hydrochlorate solution in the distractor pattern. One bout was defined as a bee leaving the nest and choosing different patterns before freely returning to the hive once she was satiated. During each bout, empty feeders were refilled with 10 μL of sucrose after the bee had left the correct pattern and made the next choice. After each bout of training and tests, patterns and feeding tubes were cleaned with 30% ethanol to exclude olfactory cues. The location and shape of all conditioned and unconditioned patterns were randomly changed before the bee could enter the arena for the next bout (Fig. 1C). Patterns and their positions were randomly varied in each bout to prevent bees from using the location of the reward when solving the task. Each day of the experiment, only one selected bee from the pre-training phase was allowed to enter the arena until a total of 12 bouts and three tests were completed. Only one of four types of patterns (big dots, small dots, large star, or small star) was presented to bee at each bout. In this study, four groups of bees were trained separately. The first group (*N* = 10) was trained to associate the pattern containing one item with a reward and to avoid the pattern with three items and the second group (*N* = 10) was trained to get a reward from patterns with three items against unrewarding patterns with a single item. The third group of bees (*N* = 10) was trained to discriminate patterns with two items over those with four items, while the last group (*N* = 10) was trained to choose patterns containing four items over two items.

To evaluate performance with novel patterns after training, the bees were examined not only in the learning test, but also in the transfer tests containing novel patterns or novel quantities (Fig. 1B, C). All patterns in the tests provided 10 μL of sterilized water (i.e., patterns without rewarding or punishing outcomes for correct or incorrect choices [ICs]). Following the learning phase, the first unrewarded test was used to determine whether bees had learnt to distinguish numbers without any olfactory and irrelevant visual cues. Also, bees were examined in transfer tests which included novel patterns (Fig. 1A, B) to assess whether bees could transfer the learned numbers to novel size, shape, or color. Finally, bees were confronted with novel quantities; patterns with two or four items were presented to bees that had previously been trained to one or three items. Conversely, bees that had learned to discriminate between two and four items were presented with patterns containing one or three items. One or two refreshment bouts of training were used between tests to maintain the bees’ motivation to complete the task. The sequence of tests was randomized from bee to bee. Trained bees were removed from the nest once the training and tests phases were finished.

### Statistical analysis

To evaluate the bees’ performance over trials, colony, groups and patterns, the data from the learning procedure were analyzed with a generalized linear model (GLM) for a binary probability of the performance. The percentages of the correct choices (CCs) were calculated for every block of 10 consecutive visits of all the bees (Supplementary Fig. S1). To study the effect of different factors on bees’ performance, we defined the trial block as a continuous predictor; colony, and the group of bees trained with patterns with different number of items were defined as categorical predictors and we included the interaction between trial block and the group of bees in the GLM. The bees’ index was included in the model to check for random effects. Finally, the GLM’s parameters were estimated by maximum-likelihood estimation method in MATLAB 2018b (MathWorks, MA, USA). In addition, the homogeneity of bees’ responses in each group was tested using Chi-square goodness of fit tests. To determine whether bees were able to extract the learnt numerical information from the training patterns without any further cues, the decision of bees during the first 120 s of their flight was analyzed in terms of choices (landing on a pattern) and rejections (hovering over a pattern and flying away without landing). This gave four possible response categories: landing on the correct pattern (correct choice - CC); landing on the incorrect pattern (incorrect choice - IC); visiting (hovering over) an incorrect pattern without landing (correct rejection - CR); visiting a correct pattern without landing (incorrect rejection, IR). The percentage of each response was estimated from the video recorded in the unrewarded test. Finally, the Wilcoxon signed rank test or Wilcoxon rank sum test was used to interpret the null hypothesis that a pair of responses was not different.

To summarize the bees’ performance in the learning and transfer tests with novel numbers, we used the modified formula for the Matthews correlation coefficient (MCC) (Matthews 1975) as follows:

$$\mathrm{MCC}=\frac{C_{l}\;\times \;R_{s}\;-\;C_{s}\;\times \;R_{l}}{\sqrt{\left(C_{l\;}\;+C_{s}\right)\left(C_{l\;}+\;R_{l}\right)\left(R_{s\;}+\;C_{s}\right)\left(R_{s}\;+\;R_{l}\right)}}$$

where $C_{l}$ and $R_{l}\;$represent the number of choices and rejections of the pattern with large number of items while $C_{s}$ and $R_{s}$ represented the number of choices and rejections of the pattern with small number of items, correspondingly. This allows a more comprehensive evaluation of choice behavior in comparison to the popular evaluation in which choice accuracy is measured by only evaluating correct and incorrect choices. This coefficient takes into account both true and false positive responses, as well as correct and incorrect rejections . MCC measures the correlation between the observed pattern of responses and the pattern of responses that would reflect perfect performance. High positive values of MCC (maximum at +1) corresponds to the tendency of the bee in responding to the patterns with large number while negative values of MCC (minimum at −1) exhibits the responses of the bee to the pattern containing small number of items. Zero indicates bees were not better than chance level to select one of the presented options. Where the previous analysis of correct versus incorrect choices indicated numerosity had been learned, we tested the directional hypothesis that MCC values were significantly greater than zero for groups trained on the higher numerosity, and significantly less than zero for groups trained on the lower numerosity (Wilcoxon signed rank test, one-tailed).

### Video analysis

**Fig. 2:**
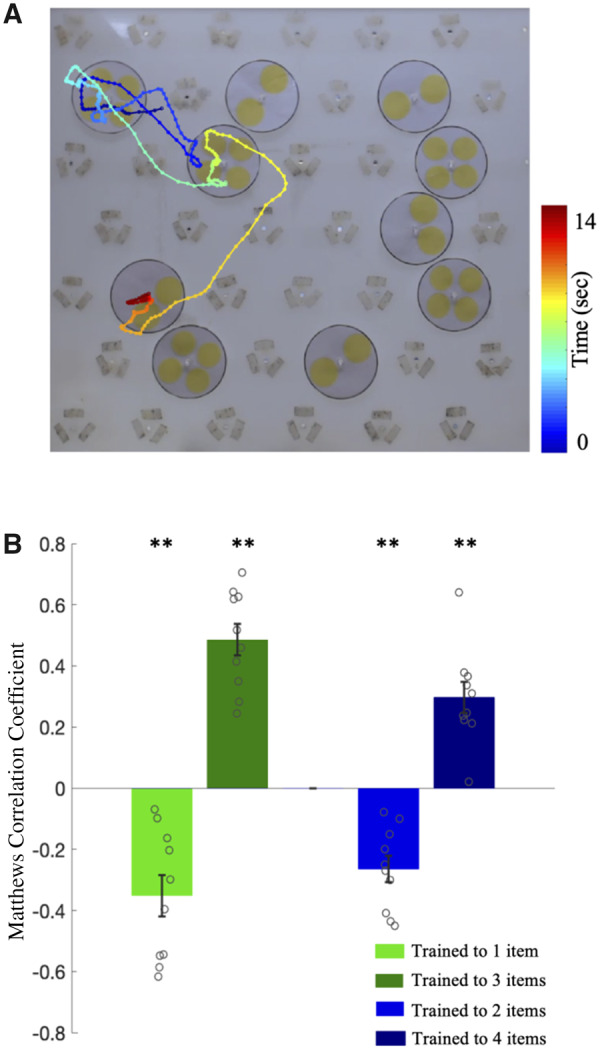
Numerosity discrimination by bees. (**A**) Flight path showing the first 14 s of activity during a learning test, from a bee trained to select two- and avoid four-item patterns. Each point on the flight path corresponds to a single video frame, with an interval of 33 ms between points. The bee sequentially scans two patterns, correctly avoiding them, before landing on a two-item pattern. The color map changes from blue to red with increasing time (see Supplementary Video S1). (**B**) MCCs (mean±SEM) for all four training groups in the learning tests. Values are indicated for each individual bee by small empty circles. The correlation is computed with respect to choosing the larger numerosity for each training group; hence, positive correlation indicates correct performance for bees trained to three- or four-item patterns (3 + 1−, 4 + 2−) while negative correlation indicates correct performance for the complementary training groups (1 + 3−, 2 + 4−). Correlation coefficients are significantly different from zero (**P < 0.001).

The arena was equipped with a camera at the top of the arena entrance (opposite the decision wall) to record the bees’ flights while they were scanning the presented patterns. The field of view of the camera was 215 cm wide and 120 cm high at a resolution of 1280 × 720 pixels (Supplementary Fig. S3). For initial trial runs with four individuals, the frame rate was 30 fps using a webcam (HD Pro Webcam C920, Logitech, Lausanne, Switzerland). We subsequently switched to 240 fps using an iPhone 5 (Apple, Cupertino, CA, USA). The first 120 s of the tests were video-recorded to analyze the bee’s scanning behavior. Examples of recorded tracks are shown in Fig. 2A and Supplementary Fig. S3 and Supplementary Videos S1 and S2.

The first 120 s of the recorded videos from the learning and quantity tests was analyzed using the free software Solomon coder beta (Andras Peter, BUDAPEST). Bees were observed to reduce the speed of flight or hover in a stable position when approaching a pattern. Hovering behavior was characterized by a bee flying very closely (approximately 1–2 cm) in front of the stimulus while facing it. We wished to quantify this behavior in a manner independent of any observer bias. Therefore, a MATLAB algorithm was developed to measure the hovering time (i.e., the time spent hovering in front a pattern), and the number of items scanned in each pattern, prior to a bee’s choice (landing) or rejection (flying away after inspection). The MATLAB algorithm was based on the extraction of *x*/*y* coordinates of the bees’ bodies in front of the target wall during flight, frame by frame. The algorithm was fully automated, allowing us to track a bee between consecutive frames, independently of target deformation, shadows, or external moving objects. Extracted flight data that represent the bees’ location at each frame of the videos was used for further analysis. The flight path of each bee was considered to start when the bee entered the arena.

We evaluated the bees’ scanning behavior depending on the final choice made (land or reject after inspection). Total hovering time in front of a pattern was as the total time the bee’s body was seen inside (in front of) the circular boundary of the pattern, from the vantage point of the camera. In a similar manner, we evaluated the number of items that bees scanned within each pattern. If the bee’s body was located entirely within the borders of an item (as viewed from the camera) for >0.2 s, we considered the bee to be scanning the item and therefore this was considered a count. Ambiguous flight movements in front of a stimulus (≤0.2 s) where bees may have been changing direction or flying across a stimulus to reach another were not considered. A simple threshold rule was used to decide if bees chose (or rejected) a target. A bee is generally considered as choosing a target when it makes contact (with antennae, feet or proboscis) with the target, and this typically involves a temporary slowing down of flight, or actual landing. Since these behaviors could not be monitored using the video material at hand in an automated manner, we chose a threshold flight speed classification to assess if bees had made contact with (chosen) a target. Since, however, flight speeds were variable between bees, we used a dynamic threshold determination. The speed of a bee in front of all microtubes was clustered into two groups using a *K*-means algorithm. The boundary between two groups (from *K*-means) was considered a threshold to identify the bee’s decisions. We assumed that the bee chose a pattern if her speed was below the threshold. Otherwise, her behavior was classified as rejection behavior.

It is possible that the criterion of counting an item as inspected only when the bee was seen right in front of it underestimates the numbers of items really viewed. For instance, the bee’s body could have been located slightly outside the volume in front of an item during the video, while the body axis was tilted in such a way that the bee may have been facing an item. In such cases, the bee may have scanned the item, but this would not have been considered a count because it did not qualify by our criteria. For this reason, we repeated the analysis for an extended volume around each countable item, so that we also counted when a bee was seen up to 5 mm outside the boundaries of the item. Measurement of hovering time and number of items scanned was done by independent analysis of all flight path data by three experimenters (HM, HSGD, and EG) and then cross-checked. The average hovering time and number of pattern items scanned were then separately calculated for each response category (i.e., CC, CR, IC, and IR; see above).

## Results

### Bumblebees discriminate numerosities in the range 1–4

We first confirmed that bumblebees could perform simple numerosity discrimination. Differential conditioning was used to train bees on artificial flower patterns containing one to four countable items (Fig. 1; see the “Methods” section). Bees were trained to discriminate one-item from three-item patterns, or two-item from four-item patterns. In each case, one group of bees was reinforced positively (+) on the higher number (3+ or 4+) and negatively (−) on the lower number (1− or 2−), while another group was subjected to the reverse conditioning. This resulted in four groups of bees (4 + 2−, 2 + 4−, 3 + 1−, and 1 + 3−). Each group was presented with five pairs of the patterns, randomly arranged on the back wall of the flight arena during training and subsequent learning and transfer tests (Fig. 1C). Before analyzing the data further, we first ensured that each group of 10 bees showed statistically homogenous behavior by means of *χ*^2^ contingency tests on the 15 sequential blocks of 10 choices per bee. Indeed all four groups were statistically homogenous (df = 126; *P* > 0.99 in all cases): 4 + 2−: *χ*^2^=29.4; 2 + 4−: *χ*^2^=17.7; 3 + 1−: *χ*^2^=27.8; 1 + 3−: *χ*^2^=20.1).

Following training, learning was assessed in unrewarded trials (learning tests) of patterns containing the same numbers of countable items. We analyzed the bees’ choice behavior using video recording of the first 120 s of activity from entering the flight arena (Fig. 2A). During this interval, bees sequentially scanned, on average, 32 patterns (Supplementary Fig. S1B); each scan led to a landing (a choice) or the bee flew on to another pattern without landing (a rejection). This yields four possible response classes: a CC, IC, CR, and IR. We counted the number of responses in each category to compute the MCC for each bee in each learning test (see the “Methods” section). In Fig. 2B, the mean MCC values are plotted for each group of trained bees (see also Supplementary Fig. S2). Since we computed the MCC as the correlation with responses to the larger numerosity in each pair of patterns, perfect performance would be reflected in a MCC of 1.0 for the 4 + 2− and 3 + 1− groups, and a MCC of −1.0 for the groups trained on the smaller numerosity (2 + 4− and 1 + 3−). In all cases a MCC of 0 reflects chance performance. The distribution of MCC values was significantly different from a distribution centered on zero for all groups (Wilcoxon one-sample signed rank test; W > 36, *P* < 0.004, two tailed, for all groups) indicating that each group successfully learned the numerosity discrimination.

Performance in the learning tests was not significantly different for bees trained on the higher or lower of the target numerosities in the patterns (i.e., 1 vs. 3 or 2 vs. 4). However, the absolute performance level was higher for groups trained to discriminate one- from three-item patterns compared with the groups trained to discriminate two- from four-item patterns, regardless of whether the target numerosity was the lower or higher value (Wilcoxon two-sample signed rank test, W = 249, *P* = 0.046). This was also reflected in the training phase, where analysis of learning curves demonstrated faster learning in the groups trained to discriminate one from three compared with the groups trained to discriminate two from four (Supplementary Fig. S1A; GLM—see the “Methods” section, *P* = 0.001).

To control for the possibility that low level visual cues may have influenced bees’ decisions, we carried out transfer tests (see the “Methods” section), in which the trained numerosities were represented by novel patterns (Fig. 1 and Supplementary Fig. S3, Supplementary Video S2, and Supplementary Table S1), where the size, shape, and color of the constituent items were varied. Most trained groups made significantly more CCs than ICs when the trained numerosity was presented via the novel patterns (Supplementary Fig. S4; *P* < 0.04 for all novel patterns and groups except the 3 + 1− group, which failed to transfer the trained numerosity to novel color patterns (*P* = 0.19), despite successfully transferring to novel sizes and shapes; see Supplementary Table S1 for full details of stimuli). We therefore conclude that bumblebees can make visual discriminations based on numerosity, at least in the range of 1–4 and when the difference between numbers was two. They did so without relying on visual pattern matching, overall area, illusory contours, spatial frequency, convex hull, or perimeter length of stimulus items. This is consistent with previous studies in honeybees (Chittka and Geiger 1995; Dacke and Srinivasan 2008; Gross et al. 2009); we first needed to ascertain that bumblebees, too, could solve such tasks before exploring *how* they solve them (see subsequent sections).

### Bumblebees scanned items sequentially

**Fig. 3:**
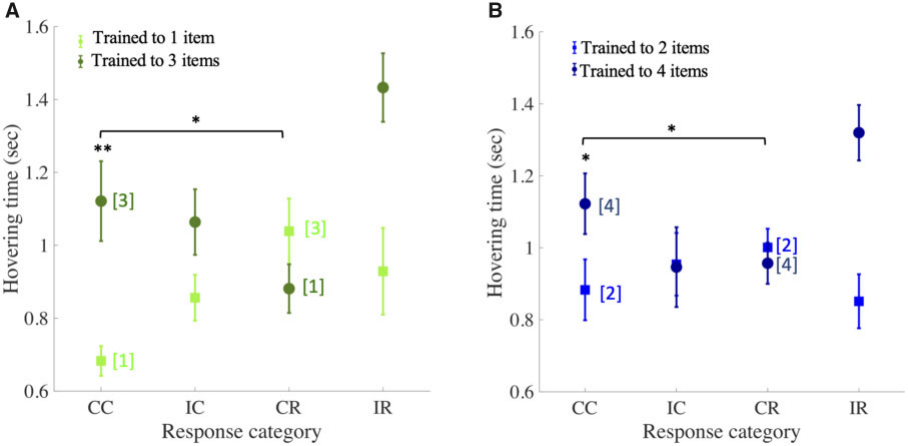
Hovering times of bees in the learning tests. (**A**) Response times (i.e., the time spent hovering in front a pattern) for each response category (CC, correct choice; CR, correct rejection; IC, incorrect choice; IR, incorrect rejection) for bees trained to select one-item (light green symbols) and three-item (dark green) patterns (mean±SEM). Numerals in square brackets indicate the numerosity for correctly chosen and correctly rejected patterns. Response time increases with pattern numerosity for both CCs and CRs. (**B**) Same analysis for bees trained to discriminate two-item (light blue symbols) from four-item patterns (dark blue symbols). ** indicates *P* < 0.001 and * for *P* < 0.05 for difference in hovering time between CC and CR within groups (horizontal square brackets) and difference in hovering time for CCs between groups.

**Fig. 4:**
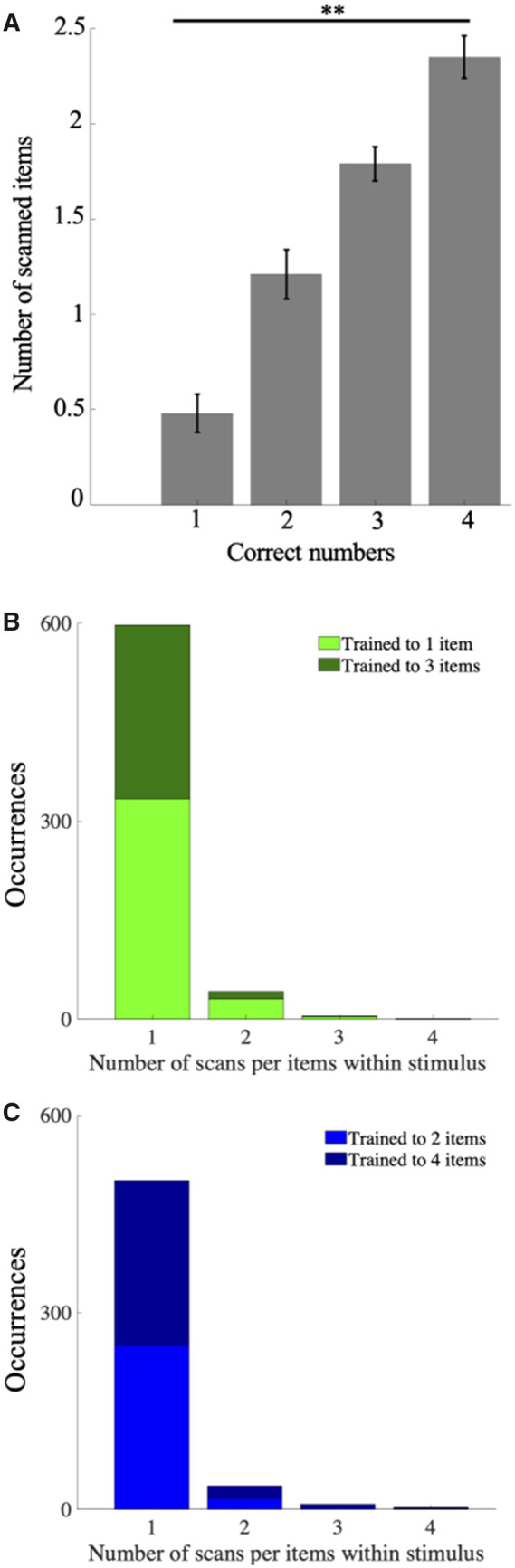
Sequential scanning of stimulus elements by bees. (**A**) Mean (±SEM) number of stimulus elements scanned prior to correct responses for four groups of bees trained to discriminate one- from three-item patterns and two- from four-item patterns. Number of items scanned increases with pattern numerosity. ** indicates *P* < 0.001 (**B**, **C**) Number of scans for each item within a pattern for bees trained to discriminate one- from three-item patterns (B) and two- from four-item patterns (C). For both groups, scanning an individual item more than once is very rare (<1% across all bees), suggesting the sequence of items scanned is retained in working memory.

To explore how bees made the choice of accepting or rejecting a given pattern, we analyzed the videos of the bees’ flight paths (see the “Video analysis” section) using an automatic extraction of the flight path (from the bee’s location at each frame of the video) and hovering time (flight duration when the location of the bee was within the circumference of the pattern followed by a “landing” or “rejecting” choice) for every pattern type. Hovering time for CCs depended strongly on training group, being significantly longer for bees trained on the larger number of items within a pattern (3 + 1− > 1 + 3−, Fig. 3A and 4 + 2− > 2 + 4−, Fig. 3B; Wilcoxon rank sum test: *P* < 0.008 for both group comparisons). In other words, bees took longer to correctly identify target patterns containing more items, presumably because such items require more scanning (Fig. 3A, B). Interestingly, a similar, if less marked effect was found for CRs (when bees found themselves scanning the incorrect number). Bees trained to patterns with the smaller number of items (1 + 3−, Fig. 3A and 2 + 4−, Fig. 3B) spent longer hovering over patterns containing the higher numerosity before correctly rejecting it, than they did over patterns containing the smaller numerosity before correctly choosing it. The opposite relationship between pattern numerosity and hovering time was found in the two groups trained on the larger numerosity: these bees were quicker to reject patterns with the smaller numerosity than they were to correctly choose patterns with the larger (Wilcoxon rank sum test: *P* < 0.02 for all four comparisons of CC with CR). Thus, response time increased with pattern numerosity for both CCs and CRs.

In keeping with this, differences in hovering time were smaller when comparing correct responses (CC or CR) to patterns with the same numerosities in different training groups. Hovering time of bees correctly choosing three-item patterns (i.e., 3 + 1− group) did not differ significantly from those correctly rejecting three-item patterns (i.e., 1 + 3− group; Fig. 3A; Wilcoxon rank sum test: *W* = 40, *P* = 0.72). The same was true when comparing CCs and CRs of four-item patterns (Fig. 3B; Wilcoxon rank sum test: *W* = 26, *P* = 0.21). However, when bees had been trained to a larger number (3+ or 4+) and encountered a pattern with a smaller number (in which case the correct response is a rejection), there was a trend for longer scanning times, as if the bees continued searching for further items. This difference was significant when comparing CCs of one-item patterns by the 1 + 3− group with CRs of the same patterns by the 3 + 1− group (*W* = 70, *P* = 0.045; Fig. 3A), but not for the same comparison of correct responses to the two-item pattern; i.e., CC by the 2 + 4− group and CR by the 4 + 2− group (*W* = 52, *P* = 0.138; Fig. 3B).

The dependence of hovering time (Fig. 3A, B) on pattern numerosity suggests that bees make their choices at least in part by sequential enumeration of items within a pattern. To confirm this, we extracted the number of items within a pattern that were scanned prior to each decision from video recordings of the learning tests. A direct comparison for CCs, of the numbers of items scanned depending on the number of items that needed to be counted within patterns, reveals a clear correlation (Fig. 4A and Supplementary Fig. S6), confirming that numerosities were not assessed by subitizing (at a glance), but instead by bees viewing the items at least in part sequentially. However, it is also apparent that the number of items counted within a pattern before making a decision is lower than the number that actually needs to be counted. There are multiple possible reasons for this (see the “Discussion” section).

Figure 4A shows that the number of items scanned increased with the target numerosity for both CCs and CRs (Kruskal–Wallis test, *χ*^2^ = 9.25, *P* < 0.001). This indicates that more scans were needed to enumerate higher numerosity patterns to correctly choose them, suggesting that bees may need to retain items scanned in working memory for enumeration, keeping track of items already scanned. Our analysis of the sequence of individual pattern elements scanned within the response time (i.e., prior to landing or flying away) supported this notion. Bees in all four training groups clearly avoid re-scanning a previously scanned item in the vast majority of cases (Fig. 4B, C).

### Transfer to novel numbers

**Fig. 5:**
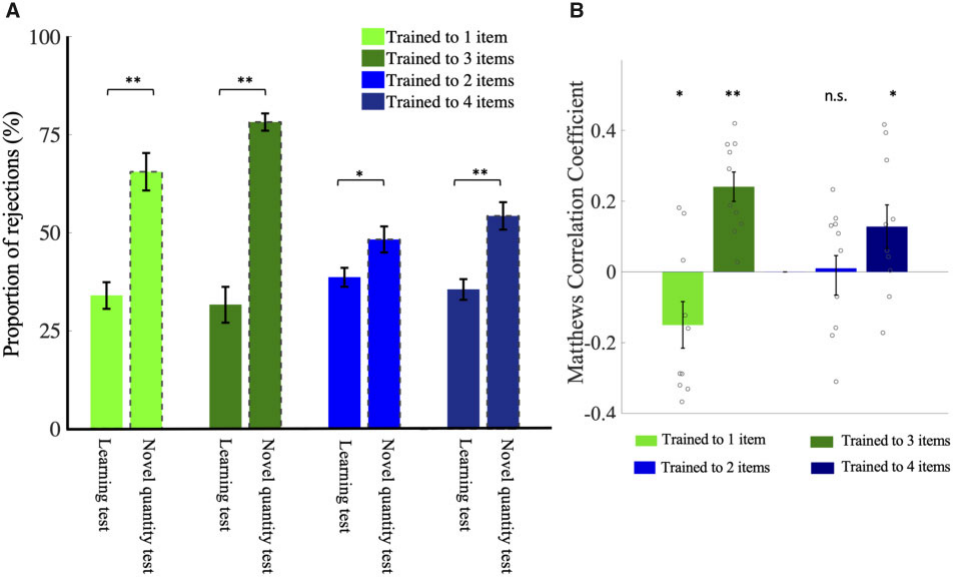
Novel numerosity test. (**A**) Comparison of rejection rate in learning test and novel numerosity tests (dashed bars) for all four training groups. **P < 0.005, *P < 0.05. (**B**) MCCs (mean±SEM; individual values indicated by small circles) for all four training groups in the novel numerosity test, where bees trained to discriminate one- from three-item patterns were presented with two- and four-item patterns, and vice versa. The correlation is computed with respect to choosing the larger numerosity for each training group; hence, if bees generalize the larger of smaller numerosity according to training group, positive correlation indicates bees trained to three- or four-item patterns (3 + 1−, 4 + 2−) are now choosing four- and three-item patterns, respectively, while negative correlation indicates the complementary training groups (1 + 3−, 2 + 4−) are now choosing two- and one-item patterns, respectively. Correlation coefficients significantly different from zero indicated by **P < 0.005 and *P < 0.05).

We next explored the behavior of the bees when confronted with novel numerosities after the training sessions. In these transfer tests, we presented each training group with pattern pairs of the non-trained pattern, i.e., two- and four-item patterns for the 1 + 3− and 3 + 1− groups, and one- and three-item patterns for the 2 + 4− and 4 + 2− groups. If the bees learned the numerical relations “greater than” and “less than” between the trained numerosities, then we would expect them to preferentially select the novel patterns containing higher or lower numerosity according to training group. When the training sessions were followed by transfer tests with novel numerosities, the overall rejection rates increased in all groups (Fig. 5A; compared with the rejections in the learning test *P* < 0.05), suggesting that the bees were reluctant to select patterns other than those containing the trained numerosity. However, analysis of choice behavior (according to the criterion that CCs mean selection of either the greater or lesser of the novel numerosities, according to training group) showed significantly more CCs among the 1 + 3−, 3 + 1−, and 4 + 2− groups. Thus, bees trained to discriminate one-item over three-item patterns were significantly more likely to select a smaller numerosity when confronted with the novel two- and four-item pattern pairs (Supplementary Fig. S5A). Conversely, bees trained to select three-item patterns over one-item patterns were significantly more likely to select the larger (four-item) pattern from the novel numerosity pair. Similarly, bees trained to discriminate four- over two-item patterns were significantly more likely to select the greater novel numerosity (three-item patterns). However, this trend was not found among bees trained to discriminate two- from four-item patterns; these bees selected the larger or smaller of the novel numerosity pairs with approximately equal frequency (Supplementary Fig. S5B).

The above evaluation took into account only landing on patterns. A similar picture emerges when MCCs (where all four types of decisions are evaluated, including pattern inspections followed by a rejection without landing) are calculated for the transfer tests (Fig. 5B). Bees of the group rewarded on three items (3 + 1−) chose four over two in the transfer test (positive correlation; *P* = 0.001), whereas bees rewarded on one-item patterns (1 + 3−) preferred two-item patterns in the transfer test (negative correlation; *P* = 0.042). Bees trained to choose two- and reject four-item patterns were indiscriminate when forced to choose between one- and three-item patterns, selecting the higher or lower of the novel numerosity approximately equally, as reflected in an MCC value not significantly different from zero (*P* = 0.56). Bees trained to four-item patterns (4 + 2−) showed only a weak trend to select three- over one-item patterns in the novel numerosity test (*P* = 0.055; Fig. 5B).

## Discussion

The aim of our study was to investigate the behavioral strategies and mechanisms underpinning counting in bumblebees. Our results suggest that bees require sequential scanning of pattern items to enumerate the countable elements within a pattern. This is supported by the observation that the time required to make number-based visual discriminations depends on quantity of items and the capacity of storing such information during inspection. We show that bumblebees can discriminate numerical quantities in the range 1–4 in a manner that rules out other low-level features that might correlate with number, at least when the difference between the countable items is two. Thus, bumblebees join honeybees and other insects in terms of their ability to respond appropriately in small-number counting tasks (Chittka and Geiger 1995; Dacke and Srinivasan 2008; Gross et al. 2009; Howard et al. 2018, 2019).

Our detailed flight-path analysis of the behavior of bees also indicates that their assessment of pattern numerosity depends on serial enumeration of pattern elements. The close-up inspection and scanning of countable items by bees was not a result of their poor detectability (see the “Methods” section). For CCs, bees trained to select the larger number of a pair would spend more time inspecting the patterns, and scan more items within a pattern, than bees trained to the smaller number. This was also true for CRs by bees trained to the smaller number of a pair, where longer hovering times and larger numbers of scanned items indicate serial enumeration of the incorrect patterns prior to rejection. Furthermore, during inspection of the patterns, the frequency of re-scanning an individual pattern item was very low (<1%), suggesting that bees maintained a tally of items scanned in working memory. This suggests that bees in our study do not make global judgments of numerical quantity, in the way that humans and other primates can (Jevons 1871; Kaufmann et al. 1949; Feigenson et al. 2004), but instead need to itemize the number of elements within a pattern, at least in part, by serial visual scanning.

Our video analysis of bees’ flight behavior was relatively simple, and has obvious shortcomings. Nonetheless, it is better to have *some* exploration of the bees’ scanning strategies in pattern discrimination tasks rather than none at all, as a first step to understanding the mechanisms behind their visual cognition. The limitations stem from the fact that only a single camera was used, and therefore we have no information about the bees’ position in 3D, and the proximity to the wall on which the targets were presented had to be estimated. In addition, the volumes in which the bees’ scanning was counted were not simple cylindrical shapes, but would instead have been slightly oblique depending on how far in the periphery of the wall the targets were. Irrespective of these shortcomings, the overall result that bees take longer to inspect patterns with larger numbers of items, and inspect (hover in front of) larger numbers of such items is unaffected by this. Therefore, the observation that bees do not fully assess small numerosities by subitizing is robust.

Nonetheless, the number of items inspected per pattern before bees make a decision is on average smaller than the number of items that need to be enumerated (Fig. 4A and Supplementary Fig. S6). Even when the larger volume around each item is taken into account, bees decided to accept a four-item pattern, on average, after scanning on average, 2.5 items. For one-item patterns, the number of items scanned is, on average, 0.5—in other words, according to our quantification, bees often landed on such patterns very swiftly and without closely scanning *any* items. There are the following possible explanations. One is that our method of diagnosing that an item was enumerated (“scanned”) are highly conservative—the bee had to spend a time of 0.2 s in front of it (or a slightly enlarged area around it). We were not able to assess if a bee might have counted an item during a shorted fly-by, or from an oblique angle. Therefore, the analyses in Fig. 4A and Supplementary Fig. S6 might present underestimations of the numbers of items scanned. It is also possible that bees viewed items by subtle head movements (Riabinina et al. 2014; Boeddeker et al. 2015) that could not be captured by our video analysis.

In addition, we cannot rule out that there was some combination of parallel or serial processing, to the extent that, for example, bees might be able to process two, but not more, items simultaneously. The observation that decisions were made after bees scanned, on average, only half as many items as were contained in a pattern, is consistent with this (Fig. 4A and Supplementary Fig. S6). This applies only, however, under the assumption that our simple video analysis really captured all instances of bees scanning an item. Furthermore, in our study, the difference between two patterns to be discriminated was always 2. This meant that bees could decide for the correct pattern earlier than enumerating the full number of countable items. For example, when two needs to be discriminated from four, a bee can make a decision after counting three items because it is clear at this stage that the pattern inspected is *not* a two.

Our findings do not exclude the possibility that, after extensive training, bees might be able to switch to rapid simultaneous assessment of quantities, as suggested (though not directly demonstrated) by Gross et al. (2009). Allowing bees to view countable items simultaneously (as opposed to Chittka and Geiger [1995] and Dacke and Srinivasan [2008] in whose studies sequential enumeration was enforced) does not mean that they necessarily count them by subitizing. Conversely, allowing bees to enumerate items sequentially (as we did here) does not decisively demonstrate that it is impossible for bees to count by parallel processing at a glance. Further experiments in which bees are precluded from scanning patterns sequentially would be desirable, for example by flashing them briefly on a screen (Nityananda et al. 2014). Alternatively, allowing bees to view targets from a distance through baffles before making a decision could also constrain the possibility to scan targets (Srinivasan and Lehrer 1988; Horridge 2000).

Our results also suggest that bees can generalize differences in trained numerosities by applying a “greater-than” or “less-than” rule, as has recently been suggested for honeybees (Bortot et al. 2019). Of the four groups of bees, three correctly chose the patterns with the larger or smaller number of items in keeping with whether they had been rewarded with the higher or lower numerosity of the original training patterns. However, when we computed MCC, this difference was only significant for two of these groups (1 + 3−, 3 + 1−). An alternative to applying greater/less than rules when faced with novel numerosities would be to base decisions on numerical proximity. Applying such a rule would explain the seemingly random choice behavior displayed by four of the bees trained to choose two- and reject four-item patterns (the trained numerosity two is equally proximate to the novel numerosities of one and three). However, the same is true of the 3 + 1− group, yet these bees chose the higher of the novel numerosities (four rather than two) at a highly significant level. Overall, our results contribute to the growing body of work showing that bees respond to continuous (Avarguès-Weber et al. 2014) and discrete quantity (Howard et al. 2018; Bortot et al. 2019) relations. Here, we additionally shed light on the mechanisms underlying these decision-making abilities.

In other animals, the upper limit of the small number system (accessible by subitizing) is around four items (Trick and Pylyshyn 1994; Hauser and Carey 2003; Feigenson et al. 2004; Agrillo et al. 2012). This system, also referred to as OFS (Feigenson et al. 2002), is accurate and capacity-limited because it essentially enumerates the number of objects that can be individuated and tracked in working memory (Trick and Pylyshyn 1994; Cowan 2001). By contrast, larger numerical quantities are thought to be processed by a separate ANS, or analog-magnitude system, where the error of the estimate scales with the quantity to be estimated according to Weber’s law. However, the existence of a discontinuity in numerical representation has been questioned; it has also been argued that representation of both countable and non-countable magnitude is characterized by scalar variability, and the apparent ease of small number recognition is simply a ceiling effect (any two numbers from the set 1–4 will have a minimum difference in Weber fraction of 25%) (Gallistel and Gelman 2000; Ross 2003). The range 1–4 of the putative small number system is within the subitizing range, within which humans can accurately count the number of items in a display “at a glance” (Jevons 1871; Kaufmann et al. 1949). However, the ability to take in a visual scene from a single sensory snapshot is a feature of the primate visual system and does not directly pertain to the question of whether there really are two separate number systems. For example, numerical displays containing many more than four items can still be estimated by humans (albeit with less accuracy) when presented very briefly, to preclude sequential counting (Burr et al. 2017).

Are the numerical abilities of bees based on an object-file system limited by working memory capacity? The scanning behavior documented here is compatible with this notion, as is also the bees’ ability to avoid rescanning the same item within a pattern (Bar-Shai et al. 2011). However, we also note that bees trained to discriminate one- from three-item displays achieved higher accuracy than the groups trained on two- versus four-items, which could also be consistent with scalar variability, given the higher ratio difference in the stimuli presented to the former groups. On the whole, however, it is clear that the bees’ counting strategy is in part sequential in nature even for small numbers, in line with other findings on limitations on parallel processing in their visual system, and the need to acquire information about visual patterns by actively scanning them (Spaethe et al. 2006; Nityananda et al. 2014; Guiraud et al. 2018). Our results appear broadly in line with the idea that numerical judgments are related to the capacity limits of storing information in working memory when performing a visual task. As recently reported by Cheyette and Piantadosi (2020), infants and primates have a lower visual memory capacity that limits their accuracy even throughout the small number range, and this might similarly apply to bumblebees.

## Author contributions

H.M. conceptualized the research, designed the experiments, performed the experiments, wrote the programs for video analysis, analyzed the data, and wrote the manuscript. H.S.G.D. analyzed the video data. E.G. performed the experiments and analyzed the video data. O.J.L., E.B., and P.D.O. performed the experiments. P.S. conceptualized the research and wrote the manuscript. L.C. conceptualized the research, designed the experiments, supervised the study, and wrote the manuscript.

## Supplementary Material

icaa025_Supplementary_Data

## Notes

From the symposium “Integrative comparative cognition: can neurobiology and neurogenomics inform comparative analyses of cognitive phenotype?” presented at the annual meeting of the Society for Integrative and Comparative Biology January 3–7, 2020 at Austin, Texas.
