# Supplementary material for: Bumblebees Use Sequential Scanning of Countable Items in Visual Patterns to Solve Numerosity Tasks
Source: Integr Comp Biol. 2020 May 5;60(4):929–42. doi: 10.1093/icb/icaa025 (PMC7750931; doi:10.1093/icb/icaa025)
Supplement: icaa025_Supplementary_Data [file icaa025_supplementary_data.zip › Maboudi et al May 2020 supplementary materials clean version.docx]

**SUPPLEMENTARY MATERIALS**

**Supplementary figures**


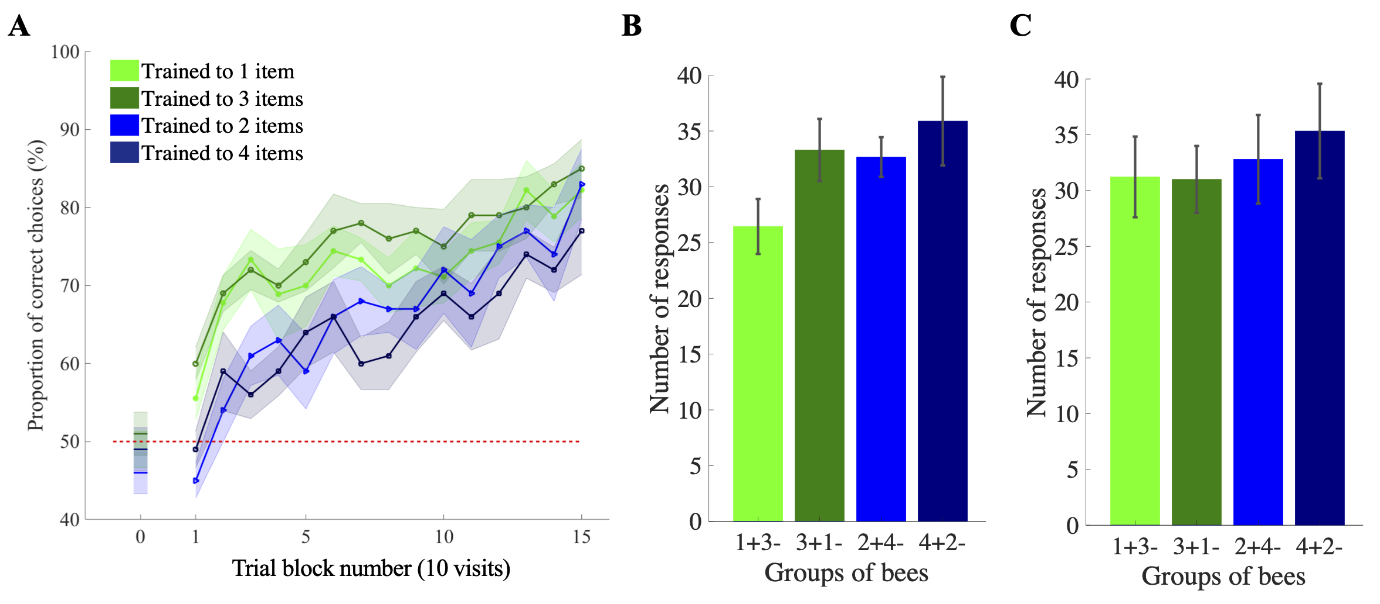


**Fig. S1. Learning curves during the training phase of the number discrimination task.** (A) Generalised Linear Model (see Methods) applied to the performance of the four groups of bees shows a significant increase in the proportion of correct choices made by the bees during the last 150 choices of the training phase (p<10^-6^), irrespective of the size, shape and configurations of items within the patterns. The mean percentage (±SEM) of correct choices are plotted as a function of blocks of 10 visits for the four groups of bees trained to patterns with two or four items (light or dark blues) and patterns containing one or three (light or dark greens). The red dashed line indicates the chance level performance. No significant differences were found between the learning curves of reciprocal groups (i.e. 1+3- versus 3+1-, and 2+4- versus 4+2- ; p=0.59). However, bees trained to discriminate between one and three items learned the task faster than those trained to discriminate two from four (p = 0.001). (B&C) Mean (± SEM) number of bees’ responses (CC, IC, CR, IR) to presented patterns in the learning test (B) and the novel quantity test (C).

**Fig. S2. Relative frequency of response categories during the learning tests in the numerosity task.** Bees trained to discriminate one- from three-item patterns (A) and two- from four-item patterns (B) chose the target numerosity (CC) more often than the unrewarding pattern (IC), and rejected the distractor numerosity (CR) more often than the target (IR). ** p<0.005, * p<0.05, n.s. p>0.05.


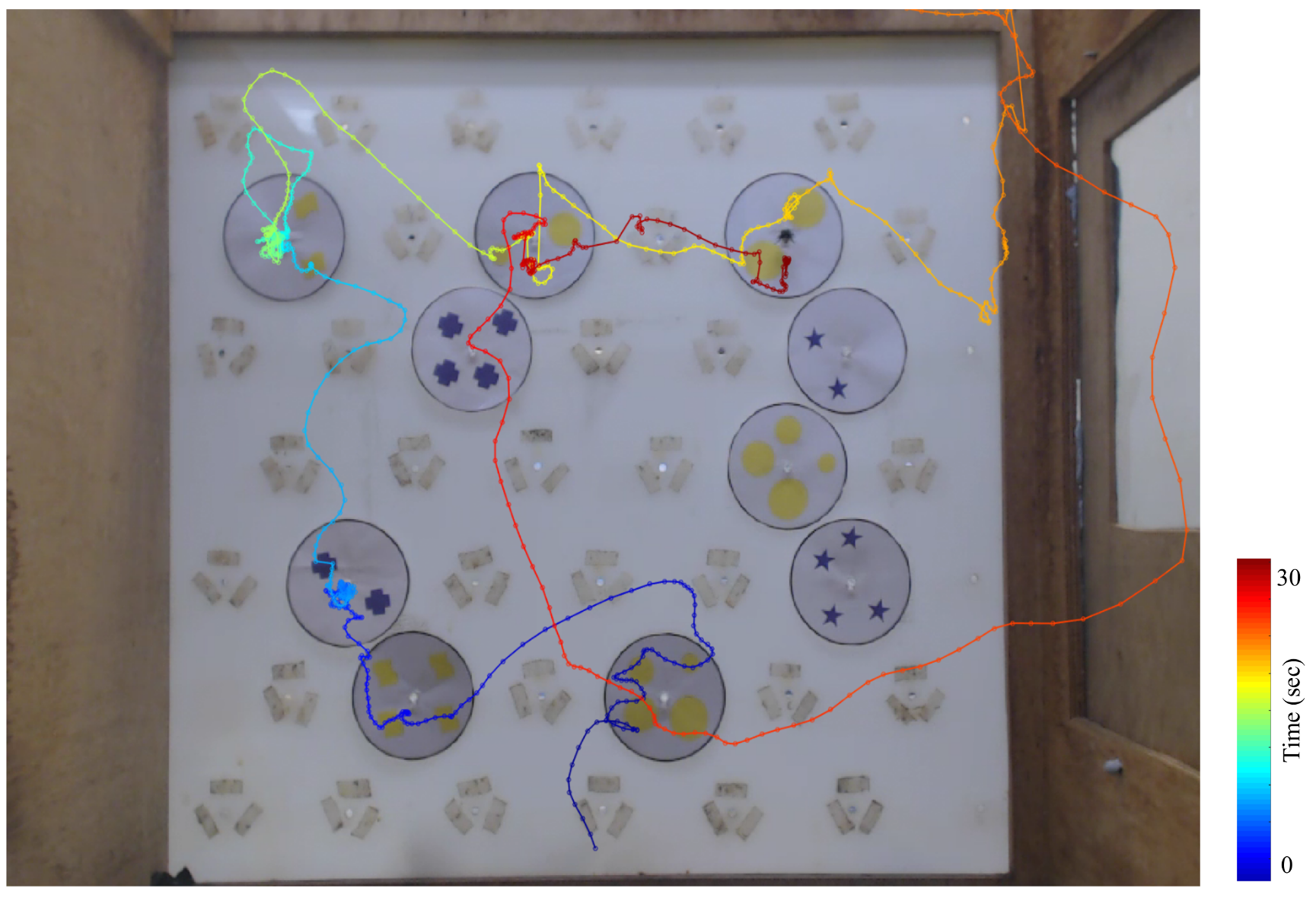


**Fig. S3. An example of the bee’s flight path in the learning test.** The bee’s scanning behaviour is colour-coded whereas dots show the bee’s location at each frame of the video with time intervals of 33 ms between frames (i.e. circles). Colours from blue to red show the bee’s locations in front of the decision wall over the time duration of flight. The bee was trained to patterns containing two items being rewarding. Following the flight, she correctly avoided the larger numbers and chose the correct pattern after inspecting items within patterns one by one. See also Videos S2.

**Fig. S4. Comparison of correct versus incorrect responses in transfer tests**. Mean percentage (± SEM) of CC (dark bars) and IC (light bars) responses for bees presented with the trained numerosities with constituent pattern elements in the form of novel shapes, colour, or size for the four training groups: 4+2- (A), 2+4- (B), 3+1- (C) and 1+3- (D). ** p<0.005, * p<0.05 except for novel colour in C (p=0.19).

**Fig. S5. Performance and scanning behaviour in the novel numerosity test.** (A, B) Percentage of response (mean ± SEM) in each category for each training group. Here, correct responses are taken as generalization of the larger or smaller numerosity according to training group to the novel numerosity pairs. For example, for bees from the 3+1- group selection of four- (CC) and rejection of two-item (CR) patterns would be considered correct. Although overall rejection rates are higher (cf Fig. 5 & Fig. S2) three of the four groups of bees chose the ‘correct’ novel numerosity significantly more often than the incorrect. ** indicates p<0.008, * for p<0.05, n.s. p=0.67.

**Fig. S6: Number of scanned items by bees during inspection before making a correct choice, depending on the correct number.** The bar graph shows the mean (± SEM) number of pattern elements scanned prior to correct responses for four groups of bees when the bee’s body was exactly in front of the items as viewed from the vantage point of the camera. Although the number of scanned items is lower than the available items within the pattern, number of items scanned increases with pattern numerosity. This approach to only count a scan when bees were seen directly in front of an item might underestimate the true number being viewed, but even when a slightly enlarged volume in front of each item is considered, the number of items tagged is still smaller than the number of items contained in each pattern (Fig 4A). ** indicates p<0.001

**Supplementary *Videos:***

**Video S1.** **Example video of the flight path of a bee previously trained to select patterns with two items.** The bee’s scanning behaviour for 14 sec are shown. This video is slowed down 2x. The bee sequentially examined items of the two patterns containing four items but rejected each of them after scanning three or four items in each. Note that she avoided the top left pattern with four items twice. She finally selected a pattern containing the correct number of two items.

**Video S2.** **Example video of a bee’s behaviour in the transfer test when faced with novel patterns.**

The video shows the bee’s scanning behaviour in front of the decision well for 45 sec when the bee was faced with non-trained patterns where the size, shape and colour of items were novel. The bee correctly rejected the first two patterns containing the larger number, after sequentially scanning the items within the patterns. she then chose the correct two-itemed patterns of novel colour and shape. Finally, the video shows how the bee continued her search for other patterns.

**Supplementary *Table:***

|  | **Patterns** | **Type of stimuli** | **Number of items** | **The edge length (cm)** | **Amount of yellow (cm^2^)** | **Area under curve of power spectrum** | **Convex hull area (cm^2^)** | **Illusionary shape** |
| --- | --- | --- | --- | --- | --- | --- | --- | --- |
| 1 | 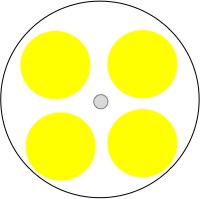 | Training | 4 | 31.41 | 19.63 | 3.13 e+8 | 26.96 | 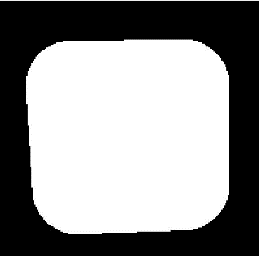 |
| 2 | 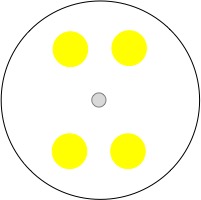 | Training/test | 4 | 15.70 | 4.90 | 6.16 e+8 | 14.04 | 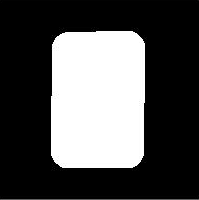 |
| 3 | 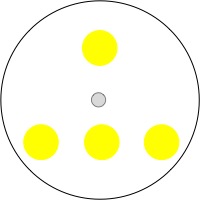 | Training/test | 4 | 15.70 | 4.90 | 6.16 e+8 | 15.50 | 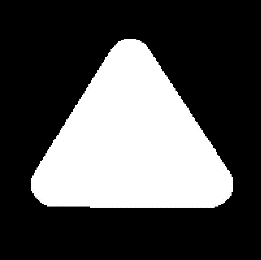 |
| 4 | 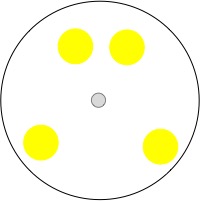 | Training | 4 | 15.70 | 4.90 | 6.17 e+8 | 19.57 | 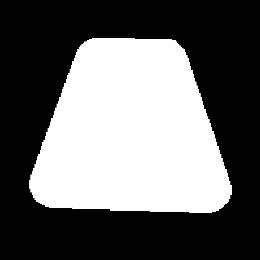 |
| 5 | 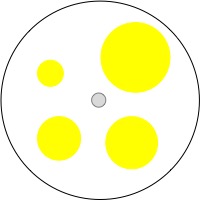 | Test | 4 | 21.99 | 11.23 | 4.90 e+8 | 19.23 | 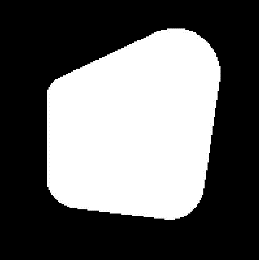 |
| 6 | 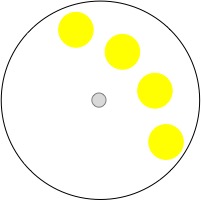 | Training /test | 4 | 15.70 | 4.90 | 6.18 e+8 | 10.33 | 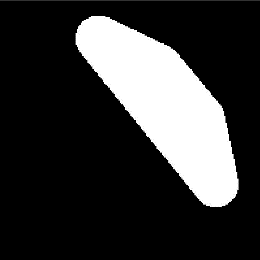 |
| 7 | 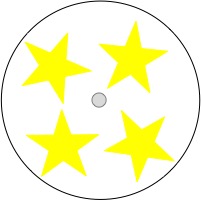 | Training | 4 | 40.00 | 7.94 | 5.34 e+8 | 25.14 | 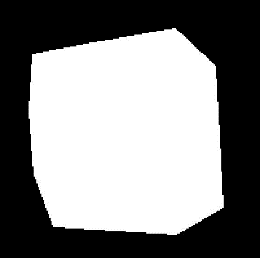 |
| 8 | 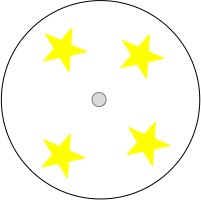 | Training | 4 | 24.00 | 3.38 | 6.60 e+8 | 20.58 | 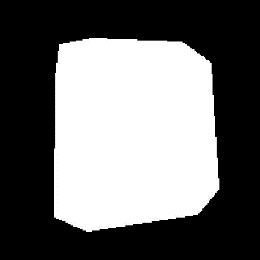 |
| 9 | 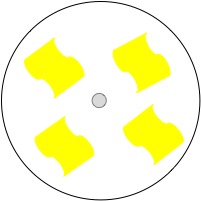 | Test | 4 | 26.40 | 9.94 | 5.17 e+8 | 22.17 | 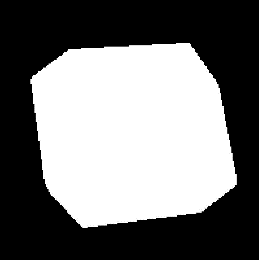 |
| 10 | 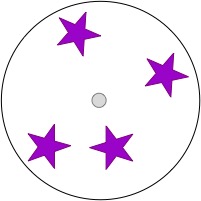 | Test | 4 | 24.00 | 3.38 | 6.41 e+8 | 21.64 | 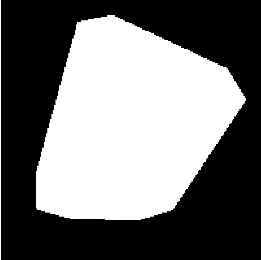 |
| 11 | 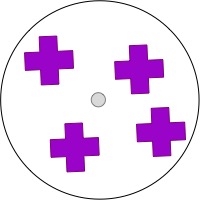 | Test | 4 | 30.40 | 9.24 | 5.38 e+8 | 20.72 | 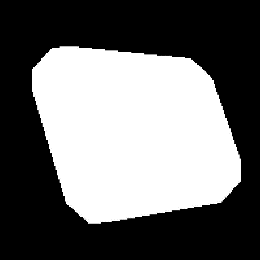 |
| 12 | 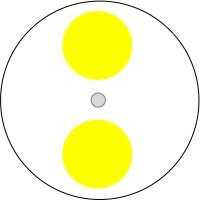 | Training/ test | 2 | 15.70 | 9.81 | 5.10 e+8 | 13.79 | 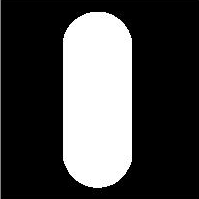 |
| 13 | 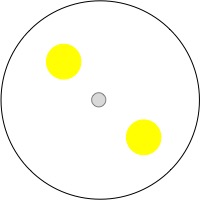 | Training | 2 | 7.85 | 2.45 | 6.81 e+8 | 5.84 | 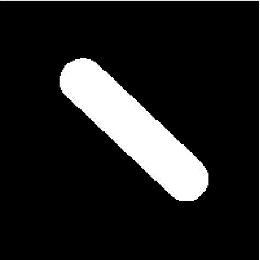 |
| 14 | 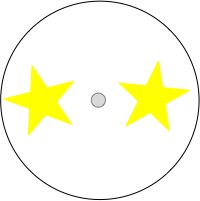 | Training | 2 | 20.00 | 3.96 | 6.38 e+8 | 12.66 | 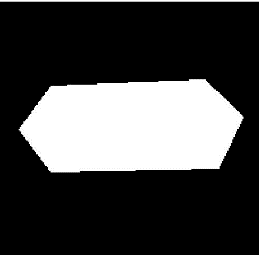 |
| 15 | 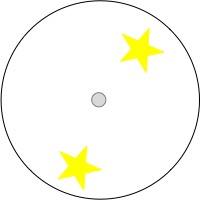 | Training | 2 | 12.00 | 1.69 | 7.04 e+8 | 8.62 | 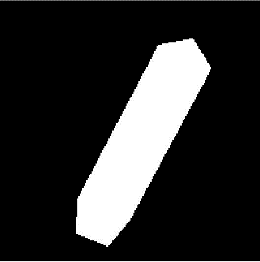 |
| 16 | 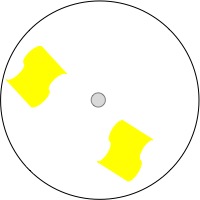 | Test | 2 | 13.20 | 4.97 | 6.29 e+8 | 9.24 | 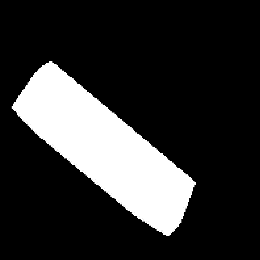 |
| 17 | 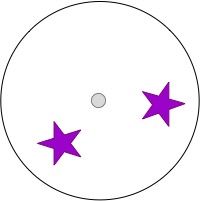 | Test | 2 | 12.00 | 1.69 | 6.94 e+8 | 6.56 | 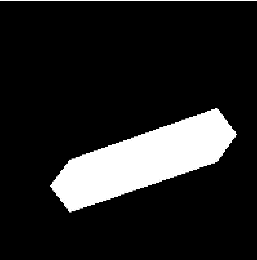 |
| 18 | 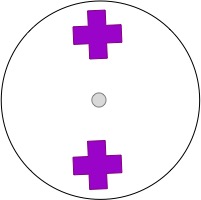 | Test | 2 | 16.20 | 3.20 | 6.41 e+8 | 9.97 | 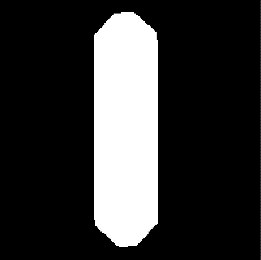 |
| 19 | 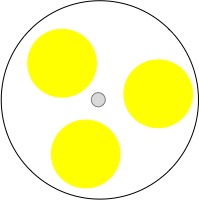 | Training | 3 | 23.55 | 14.72 | 4.07 e+8 | 21.11 | 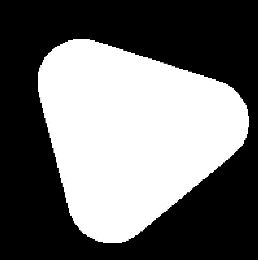 |
| 20 | 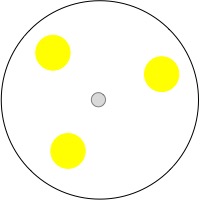 | Training | 3 | 11.77 | 3.68 | 6.49 e+8 | 14.08 | 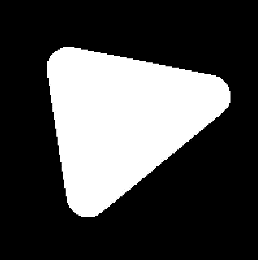 |
| 21 | 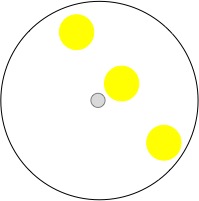 | Training | 3 | 11.77 | 3.68 | 6.50 e+8 | 7.00 | 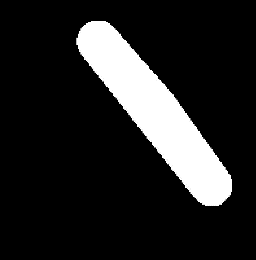 |
| 22 | 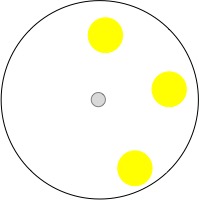 | Training | 3 | 11.77 | 3.68 | 6.50 e+8 | 11.59 | 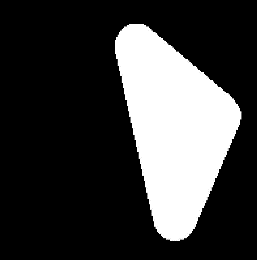 |
| 23 | 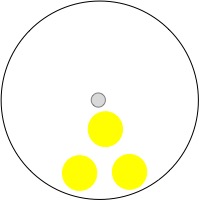 | Training | 3 | 11.77 | 3.68 | 6.50 e+8 | 5.42 | 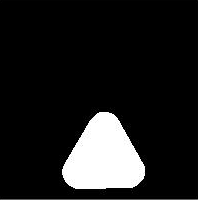 |
| 24 | 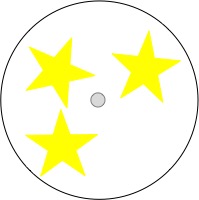 | Training | 3 | 30.00 | 5.94 | 5.86 e+8 | 18.06 | 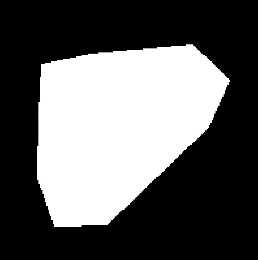 |
| 25 | 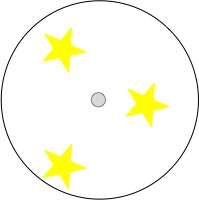 | Training | 3 | 18.00 | 2.53 | 6.82 e+8 | 13.96 | 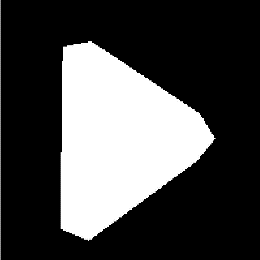 |
| 26 | 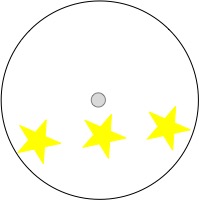 | Training | 3 | 18.00 | 2.53 | 6.82 e+8 | 7.13 | 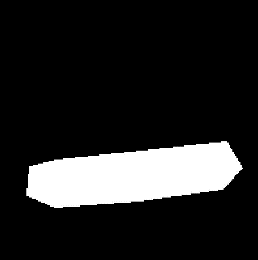 |
| 27 | 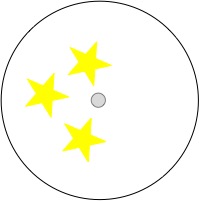 | Training | 3 | 18.00 | 2.53 | 6.82 e+8 | 6.69 | 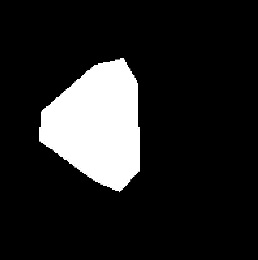 |
| 28 | 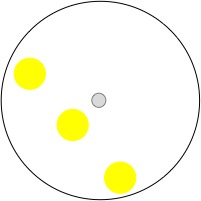 | Test | 3 | 10.16 | 3.68 | 6.67 e+8 | 5.74 | 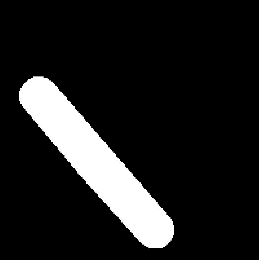 |
| 29 | 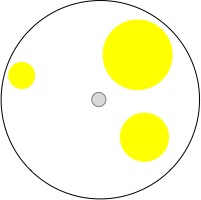 | Test | 3 | 16.33 | 9.06 | 5.45 e+8 | 17.27 | 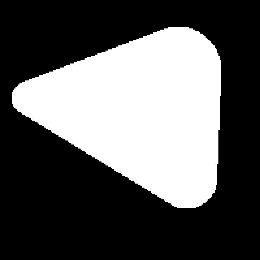 |
| 30 | 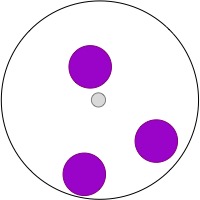 | Test | 3 | 14.13 | 7.24 | 5.91 e+8 | 14.37 | 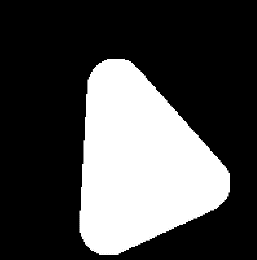 |
| 31 | 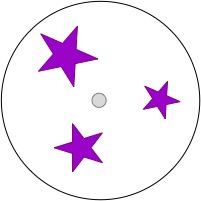 | Test | 3 | 21.00 | 5.02 | 6.48 e+8 | 14.78 | 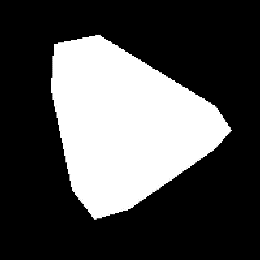 |
| 32 | 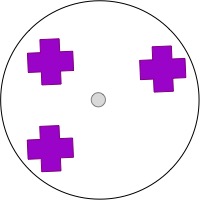 | Test | 3 | 20.40 | 7.24 | 5.90 e+8 | 17.58 | 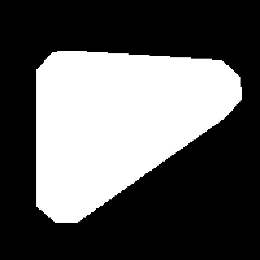 |
| 33 | 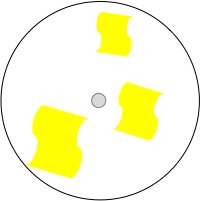 | Test | 3 | 20.4 | 8.56 | 6.57 e+8 | 16.43 | 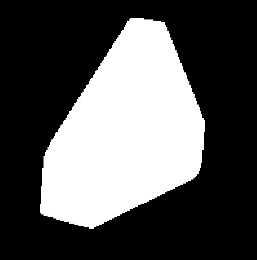 |
| 34 | 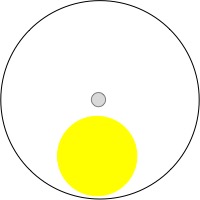 | Training | 1 | 7.85 | 4.90 | 5.86 e+8 | 4.90 | 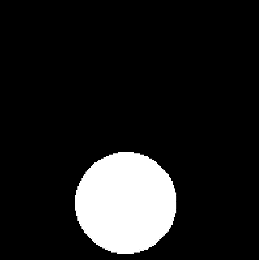 |
| 35 | 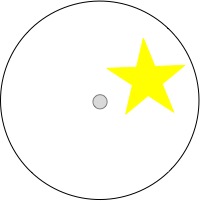 | Training | 1 | 10.00 | 1.98 | 6.81 e+8 | 4.18 | 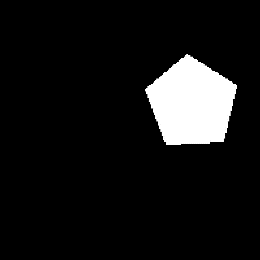 |
| 36 | 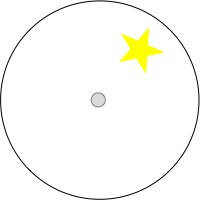 | Training | 1 | 6.00 | 0.84 | 7.26 e+8 | 1.07 | 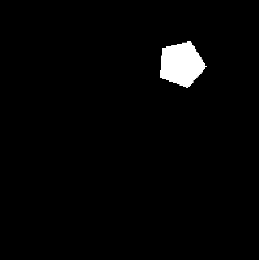 |
| 37 | 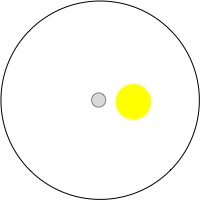 | Training/test | 1 | 3.92 | 1.22 | 7.14 e+8 | 1.22 | 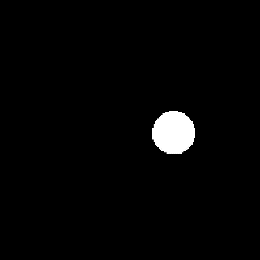 |
| 38 | 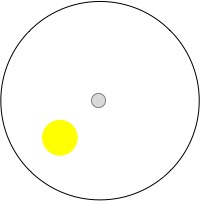 | Training | 1 | 3.92 | 1.22 | 7.16 e+8 | 1.22 | 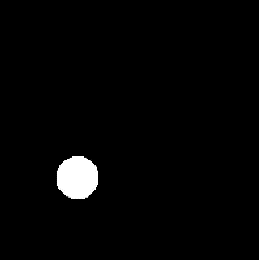 |
| 39 | 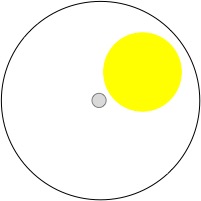 | Test | 1 | 8.48 | 5.83 | 5.92 e+8 | 5.83 | 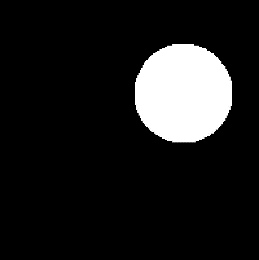 |
| 40 | 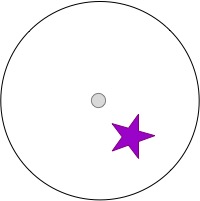 | Test | 1 | 6.00 | 0.84 | 7.23 e+8 | 1.36 | 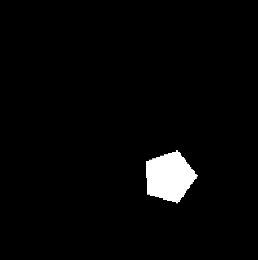 |
| 41 | 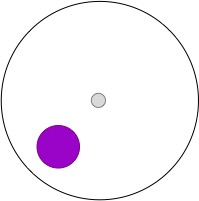 | Test | 1 | 4.71 | 1.71 | 6.34 e+8 | 1.71 | 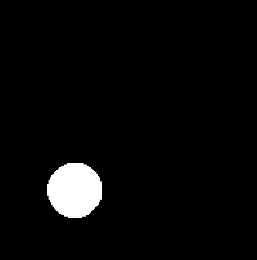 |
| 42 | 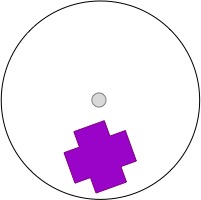 | Test | 1 | 10.60 | 3.81 | 6.96 e+8 | 4.48 | 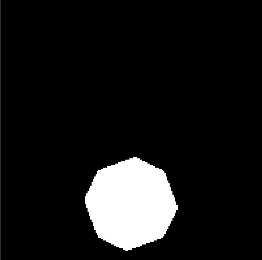 |
| 43 | 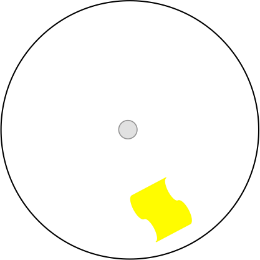 | Test | 1 | 5.40 | 1.26 | 7.06 e+8 | 1.45 | 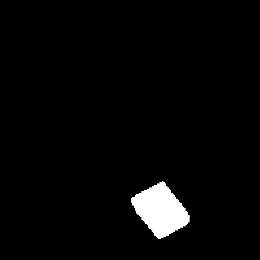 |

**Table S1:**

**Physical properties of the presented patters in the numerosity experiment.**

The second column exhibits the 43 patterns used in the training procedure and/or in the transfer tests. The third and fourth columns specify the type of the patterns and the number of items within the patterns. The fifth and sixth columns show the total edge length and the total amount of the colour of all items used in the patterns respectively. The seventh column displays the area under curve of the power spectrum that denotes the amount of spatial frequency of the patterns in the second columns. The eighth column shows the convex hull area of the items in the pattern as the minimal convex region covering all items. The last column illustrates the illusionary shape obtained from different arrangements of the items within the pattern. The table indicates that patterns with different number of items contain different low-level visual cues. Note that all training and test patterns were rotated around the centre during the experiment.
